# Bioinformatics in translational drug discovery

**DOI:** 10.1042/BSR20160180

**Published:** 2017-07-07

**Authors:** Sarah K. Wooller, Graeme Benstead-Hume, Xiangrong Chen, Yusuf Ali, Frances M.G. Pearl

**Affiliations:** School of Life Sciences, University of Sussex, Falmer, Brighton BN1 9QJ, U.K.

**Keywords:** computational biochemistry, drug discovery and design, genomics

## Abstract

Bioinformatics approaches are becoming ever more essential in translational drug discovery both in academia and within the pharmaceutical industry. Computational exploitation of the increasing volumes of data generated during all phases of drug discovery is enabling key challenges of the process to be addressed. Here, we highlight some of the areas in which bioinformatics resources and methods are being developed to support the drug discovery pipeline. These include the creation of large data warehouses, bioinformatics algorithms to analyse ‘big data’ that identify novel drug targets and/or biomarkers, programs to assess the tractability of targets, and prediction of repositioning opportunities that use licensed drugs to treat additional indications.

## Introduction

Recent estimates suggest that it takes approximately 13 years and a ‘capitalized’ cost of approximately US$1.8 billion to bring a new drug to the market [1]. This cost includes the development of the licensed drug, and also incorporates the cost of the compounds that failed to make it to the market. Projects can fail in all the different steps of drug discovery process and in particular, during the later stages of development.

Common reasons for this high attrition rate include lack of clinical efficacy of the potential drug (approximately 30%), unexpected toxicities (>20%) as well as the inherent commercial concerns (>20%) of being able to successfully position a new drug within a competitive market [2].

**Figure 1:**
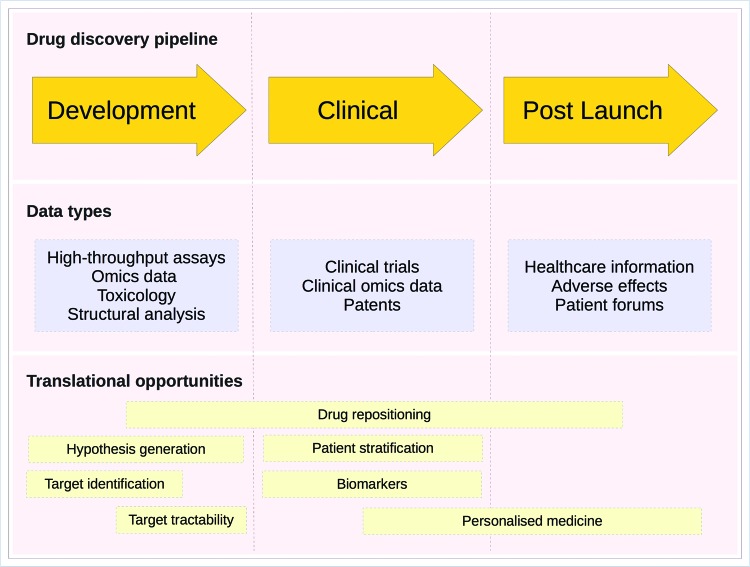
Translational bioinformatics opportunities in the drug discovery pipeline A schematic diagram of the drug discovery process. Each phase of the drug discovery pipeline (discovery, clinical and postlaunch) is shown as an orange arrow. Underneath the pipeline, shown as blue rectangles, are the types of ‘big data’ that can be generated in each step of the pipeline. Highlighted below the data types are the potential opportunities to improve the pipeline using bioinformatics techniques. For example, during the discovery phase, the focus is on identifying the druggability of potential target proteins. During the clinical trials, phase personalized medicine and patient selection can be used to better sample and categorize subjects while the use of biomarkers can improve efficacy measurements. Finally, at the post-launch phase of a drug’s life cycle drug safety monitoring and disease subtyping can be used to both improve the quality of life for patients as well as help to identify the opportunities for modified interventions that may be more effective for certain subtypes of a given disease. Adapted from [4], Copyright (2011), with permission from Elsevier.

Reducing costs and amount of time required for each of the different steps in the drug discovery pipeline is the key to deliver better drugs to patients in a timely manner [3]. One approach that has the potential to increase the efficiency of the drug discovery process involves maximizing the information acquired from the basic science. Translational drug discovery involves the effective conversion of advances in basic biological and chemical science research into the production of new drugs and treatment options for patients, i.e. the development of new drugs from ‘bench-to-bedside’. Translational approaches also come with the additional benefits of enabling new treatments and research knowledge to reach the patient subpopulations for whom they are intended, inform better clinical trial design, and help to reduce the often severe side effects of treatments. Figure 1 sets out the steps of the process and the bioinformatics techniques that can be brought to bear on them.

In this review, we illustrate how the recent advances in computational methods, together with ever growing access to publicly available medical big data are revolutionizing translational drug discovery, resulting in the development of better drugs and therapies. This revolution is happening both from the clinical perspective of disease or its pathology the ‘disease-based’ approach and from a chemical perspective, the so-called ‘drug-based’ approach.

## Disease-based bioinformatics approaches

Disease-based bioinformatics approaches in translational drug discovery are dependent upon the type of disease under consideration, with different strategies implemented to analyse cancer, genetic and infectious diseases [5].

Cancer cells are characterized by a diverse set of genetic and epigenetic changes, and by chromosomal instability. Bioinformatics approaches can be used to identify the key drivers of cancer in each particular patient. So, they have the potential to enable a more personalized approach to cancer therapy, paving the way for novel and repurposed drugs that target specific proteins, killing or disabling just those cells that are diseased [6,7].

Our genetic makeup affects our likelihood of developing a wide range of diseases, our responses to a variety of drug treatments and the progression of many infectious diseases [8–11]. For genetic diseases, the emphasis of bioinformatics techniques is often on identifying opportunities for gene therapies, as well as identifying noninvasive diagnostic and prognostic tools.

Bioinformatics is also implemented within translational drug discovery in infectious diseases. For instance, the presence of viral or bacterial infection gives rise to specific profiles of gene expression within the cell. Comparing these profiles with those of other diseases and with drug-induced genetic profiles offers repositioning opportunities for existing drugs [12–16].

### Target identification in cancer

Since the human genome was first sequenced, genomic, proteomic and metabolomic high-throughput platforms have increasingly allowed analyses of large datasets across many different diseases. Data science, machine learning and/or statistical approaches are used to identify abnormal patterns that correlate with the disease process, often with the ultimate aim of identifying actionable targets that are druggable [3].

Over 200 forms of cancer have been described [17]. Each involves dynamic changes in the genome, including a wide range of different genetic aberrations such as somatic mutations, copy number variations, as well as changes to gene expression profiles, and different epigenetic patterns. Not only do these anomalies vary among cancers, but there is also a significant variation within patient cohorts within the same cancer, with continuing changes as tumours evolve, for instance when tumours develop resistance to specific drugs [18]. The complexity of these changes means that the application of bioinformatics techniques is often critical in identifying the type of cancer presented, with each characterized by a different molecular profile that requires a unique therapeutic strategy.

Within the field of cancer research, there are several large repositories storing multiplatform cancer data including the International Cancer Genomics Consortium (ICGC) [19], the National Cancer Institute Genomics Data Commons (GDC) and The Cancer Gemome Atlas (TCGA) [17]. For example, the GDC [20] provides curated storage for over 14,531 cases previously curated by the TCGA [17], and this is expected to grow to over 30,000 cases with the inclusion of data from Foundation Medicine Inc.. The benefit of such resources is not only the access to raw sequencing data, but also the application of state of the art methods for generating high level data (e.g. mutation calls, structural variants etc.), that allow the first steps of analysis to be standardized for reproducibility as well as clinical data. It provides access to multiple ‘omic’ data types such as mRNA expression, somatic mutations, copy number variation and protein abundance.

Drawing this information together provides a better molecular characterization and understanding of the biological basis for diseases. The use of such ‘big data’ to search for novel drug targets splits into several key elements, including identifying the genes that are driving the cancer, and then determining which of these are actionable.

#### Identification of genes that may be driving cancer

Within any tumour, only a minority of the genetic changes enable and drive the progression of the disease. The other mutations provide no growth advantage and are often described as passenger mutations. Vogelstein et al. [21] identified approximately 140 potential genes that act as drivers of tumorigenesis. However, as the number of analyses grow so does the list of potentially significant driver genes [22].

A number of methods have developed to separate true driver genes from the more commonly mutated passengers. One approach (e.g. MutSigCV [18]) is to modify the putative mutation background rate to take in account the replication time of the DNA region and incorporating information about gene expression levels. In cancers with particularly high mutation rates, most genetic changes are incidental to the development of the cancer, so it is helpful to assess the functional impact of modifications. There are a number of existing algorithms that can help with this and these are outlined in Table 1. These methods use a variety of approaches to predict the tolerance of amino acid substitutions (or indels) within the protein [23] and several were specifically developed to assess the importance of particular missense mutations within cancer samples [23]. An alternative, powerful approach is to look at the events in a given genetic pathway. Results from such studies can be easier to interpret as they may suggest causal mechanisms relating to concepts such as inflammation or DNA damage response [24–26].

**Table 1:** Bioinformatics resources to help identify the functional impact of mutations and tools designed to analyse cancer mutations*

Tool	Reference	Comments	URL
CHASM*	[27]	Probability that the mutation gives the cells a selective survival advantage	http://wiki.chasmsoftware.org/index.php/Main_Page
Condel	[28]	Combines FATHMM, mutation assessor etc.	http://bg.upf.edu/fannsdb/
FATHMM*	[29]	Distinguishes between cancer promoting and ‘neutral’ germline polymorphisms using hidden Markov models	http://fathmm.biocompute.org.uk/about.html
Mutation assessor*	[30]	Based on evolutionary conservation of the affected amino acid in protein homologues	http://mutationassessor.org/r3/
Polyphen-2	[31]	Uses straightforward physical and comparative considerations	http://genetics.bwh.harvard.edu/pph2/
SIFT	[32]	Based on sequence homology and the physical properties of amino acids	http://sift.bii.a-star.edu.sg/
TransFIC*	[23]	Transforms functional impact scores provided by other tools by taking into account the differences in basal tolerance to germline single nucleotide variants (SNVs) of genes that belong to different functional classes	http://bg.upf.edu/transfic/home

#### Targeting oncogenes and tumour suppressor genes

Bioinformatics can not only help to identify genes that may drive cancer, but can also help to classify them, according to whether they must be activated (proto-oncogenes) [33] or alternatively inactivated (tumour suppressor genes) before they cause harm. The patterns of mutations seen in these two classes of genes differ considerably and have been used to separate genes between these classes when the biological function of the protein product of the gene in a cancer setting is still unknown [34–36].

Many targeted anticancer drugs work by directly inhibiting activated oncogenes, particularly proteins that contain protein kinase domains or proteins that are nuclear receptors [33,37,38]. For example, dabrafenib has been approved for the treatment of late-stage melanoma, and targets the constitutively activated kinase oncogene BRAF V600E. Whereas cetuximab, panitumumab, gefitinib and erlotinib are the licensed inhibitors of the EGFR tyrosine kinase and crizotinib is an ALK inhibitor, all of which are licensed for the treatment of lung cancer [39–42].

A substantively different approach is needed to provide therapies aimed at controlling the damage done by inactivated tumour suppressor genes. It is not usually feasible to repair the protein products of these genes, if they are inactivated by truncation, although there are on-going attempts to reactivate or restore function to a small subset of p53 missense mutant proteins [43]. While targeting a tumour suppressor gene, it is now becoming common to look for a synthetically lethal partner gene that can be drugged. Two genes are said to be synthetically sensitive or lethal (SSL) if the function of either gene can be disrupted without causing cell death, while alterations in both genes cause cell death [44]. By drugging synthetic lethal partners, it is possible to target only those cells that have the mutation while leaving normal cells viable [45,44].

Genes involved in the DNA damage response are prime candidates for synthetically lethal interactions as there are multiple complementary pathways for repairing DNA [46]. The best example of the therapeutic exploitation of SSLs is the pharmaceutical inhibition of PARP1 [47], a key enzyme in single-strand break repair (SSBR), which is SSL with genetic defects in the BRCA1, BRCA2 or PALB2 homologous recombination (HR) proteins observed in hereditary breast, ovarian, pancreatic and prostate cancers. The furthest progressed PARP inhibitor, olaparib (AZD-2281), was approved by the EMA and the FDA in late 2014 for BRCA-mutated advanced ovarian cancer patients [48] and is in further clinical trials for a variety of other SSBR-deficient cancers.

### Targeting genes in genetic disorders

Genetic disorders are generally caused by genetic variants that ultimately induce a detrimental change in protein function within the cell. Genome-wide association studies (GWAS) are undertaken to statistically associate the presence of particular genetic variations with the onset of disease. Early GWAS, based on linear regression models, were successful at identifying Mendelian traits [49–52] and disorders that are highly heritable such as coeliac disease [53] and type-1 diabetes [54,55].

Gene therapies offer a potential way to translate the results from GWAS into new treatments. Early successes were reported in 2000 for a gene therapy to treat X-linked severe combined immunodeficiency (SCID-X1). However, other gene therapy trials were placed on hold following cancers caused by insertional mutagenesis associated with the gene vectors used. More modern vectors have improved safety features, new trials have started [56], and ADA-SCID gene therapy was endorsed by the European Medicines Agency in June 2016 [57].

GWAS are now frequently employed to identify rare variants that contribute to multifactorial diseases, but it is harder to identify the relevance of their significance. This is because there are complex confounding factors in the relationships among individuals, and the relationships among the mutation loci [58,59]. GWAS have not directly identified the existing drug targets for a disease. However, Cao and Moult [60] suggest that new targets will be discovered using GWAS, by combining the techniques with protein interaction network data and machine learning.

Increasingly, the data from GWAS are being used as the starting point for a variety of different machine learning techniques. This work has potential within the clinic to provide noninvasive diagnostic tools. For example, diagnosis of coeliac disease traditionally requires exposure to the allergen gluten. However, Abraham and Inouye [54] used genetic profiling to enable the noninvasive prediction of coeliac disease without recourse to gluten sensitivity testing. Results from 1390 GWAS have been brought together and re-annotated to provide GRASP, a database of over 6.2 million SNP–phenotype associations [61].

Very occasionally, it is also possible to find the deletion of a gene that is associated with unusually good health. The 2010 Longevity Genes Study enabled Barzilai et al. [62] to study the relationship between gene polymorphisms and age. In particular, longevity was found to be associated with a deletion at in the adiponectin (*ADIPOQ*) gene.

### Infectious diseases

There are many infectious diseases that have no effective treatment or where treatment is only effective for a subset of the patient population. Moreover, variants of diseases continue to emerge, threatening the progress already made [63].

Several bioinformatics approaches have been used to stratify the patient populations. For example, GWAS have enabled researchers to identify subpopulations that have genetic variants associated with different patterns in disease progression [63,64]. Alternatively, it is possible to map the gene expression profile that is associated with disease and compare it with pre-existing profiles that are associated with drugs [65].

In 2015, a large GWAS by the Malaria Genomic Epidemiology Network found that approximately 33% protection against severe malaria is provided by genetic variants at a novel genetic locus, which is either in or close to genes encoding the production of glycophorins [66]. Therapies for infections such as HIV have been developed that target host factors [67] and it is now hoped that the same approach can be taken to improve therapies for malaria [68].

As well as enabling identification of differences between patients, ‘omic’ data can be used to identify distinguish related strains of viruses and bacteria, both by looking at evolution of the pathogen genomics, and by looking for changes in the metabolites that they express. For example, the variants of *Escherichia coli* found in the gut and urinary tract differ in the expression of two small molecules, yersiniabactin and salmochelin, that are known to support bacterial survival. Targeting the metabolic pathways or the strains that produce these molecules may provide a good strategy for preventing recurrent urinary tract infections [69]. Fontana et al. [70] provided a useful overview of this large and growing area.

‘Omic’ data also provide a fast and cheap way of identifying drugs that have potential for repurposing. The publicly available Connectivity Map allows easy comparison of any gene expression profile against the expression profile generation by over 1300 compounds, most of which are drugs that have already been approved for other purposes. The program calculates a connectivity score, an assessment of the positive or negative correlation between gene expression signatures [13]. The later DMAP extends this search to over 289,571 chemical entities [71]. In 2010, Josset et al. [14] identified antiviral agents that are broadly effective against influenza A, a virus noted for its genetic diversity. They reasoned that a viral infection could be treated by manipulating the cell environment away from the optimal conditions required for the viral life cycle [14]. This approach also has the potential to identify candidate small molecules to reverse or prevent the biological responses induced by ZIKV infection, which could have therapeutic benefits for ZIKV-infected individuals [15]. Alternative approaches looked for similarities between two diseases or two drugs by comparing the induced gene expression profiles [16].

## Drug-based approaches

### Drug repositioning and open source drug discovery

Repositioned drugs, in which the preclinical and safety studies in humans have already been evaluated, enable a faster, cheaper and more efficient translation into the clinic [72]. The use of an existing drug for a new condition is not completely risk free and still requires a drug development phase [72,73]. However, repositioning an already licensed drug can reduce the drug development cycle from 10 to 17 years to as short a time frame as 3–12 years [74].

Iorio et al. [33] mapped cancer-driven alterations on to human cancer cell lines allowed sensitivity testing with 265 existing drugs. The result of this work is the identification of a series of alterations that result in sensitivity and resistance to particular drugs, providing datasets that can act as a resource for researchers looking for therapeutic options for particular cancer subpopulations.

Data sharing, focused around a particular disease or a group of diseases, can also improve the efficacy of drug discovery and allow links to be made to other related conditions. For example, the Malaria Box provides open access to information on safety and effectiveness of compounds that kill malarial parasites *in vitro*, encouraging collaboration between academia and industry [75]. The resulting drug development programmes suggest that some of the compounds may have much wider therapeutic benefits against other pathogens and have led to the development of a wider initiative–the Pathogen Box [66]. A similar approach was taken by the TDR targets database that provides data and predicted druggability relating to tropical disease pathogens [76].

### Target tractability

Bioinformatics techniques are also used to assess whether a target is ‘druggable’. By carrying out such analyses in the early stages of drug discovery, it is possible to reduce the risk of project failure later on in the discovery process [77,78].

#### Ligand-based druggability

Protein druggability is defined as the protein’s ability to bind small drug-like molecules with high affinity. These interactions depend strongly on both ways in which the protein is folded in space and other physical attributes of the protein such as the distribution of charge. The structure of the small ‘drug-like molecule’ is equally important. An ideal drug should be able to be orally administered in small quantities. Thus, as well as being potent, the drug should successfully cross both the intestinal and cell membranes, be transportable through the blood, diffuse quickly and excreted successfully. Potential drugs that do not have these pharmacokinetic properties are a big factor of overall attrition rates [79]. These properties are well expressed and quantified in Lipinski’s ‘rule of 5’ (see Table 2) [80], and this can be improved by putting in place further restrictions on the polar surface area (PSA) and the number of rotatable bonds [81].

**Table 2:** Properties of small molecule drug-like compounds

**Lipinkski’s rule of 5** [80]
- Molecular weight ≤500
- logP ≤5
- Hydrogen bond donors ≤5
- Hydrogen bond acceptors (all N and O atoms) ≤10
**Further considerations** [81]
- PSA ≤140 A^2^
- Rotatable bonds ≤10

Introduced in 2002, the concept of the ‘druggable genome’ identified the genes within the human genome that coded for proteins that could be modulated by small drug-like proteins [82]. This bioinformatics analysis evaluated the ‘druggability’ in all human proteins by calculating their sequence identity to known therapeutic targets and predicted that less than 10% of the human proteome was druggable [82]. Of these targets, only 10% are then associated with an FDA-approved drug. The Illuminating the Druggable Genome (IDG) program aims to provide comprehensive access to data on these protein targets in order to stimulate research [38].

The ability of a protein to bind drug-like compounds can be assessed by analysing the chemical qualities of known inhibitors or predicted through virtual screening and docking of inhibitors on these proteins [83]. The CanSAR database [84] provides a ligand-based druggability scores for human proteins estimated from the chemical properties and bioactivity parameters of small molecule compounds deposited in the ChEMBL database [85]. The score is derived from the affinity, diversity, ligand efficiency and other qualities of all compounds tested against both the target and all its family members [84].

Where enzymes have similar ligand-binding profiles, this can indicate that they have similar function. For example, the family of cytochrome P450 enzymes (P450s) play important roles *in Mycobacterium tuberculosis*. Using fragment screening, Kavanagh et al. [86] identified similarities in the ligand-binding profile of CYP121A1, which is known to be important for *M*. *tuberculosis* viability, and the orphan enzyme CYP144A1. An assessment of the similarities and differences in binding between the two enzymes provides insight into both the function of the enzyme and potential inhibitors [86].

Hajduk et al. [87] experimentally showed that the druggability of a binding site is related to its ability to bind small ligands. The same principles were then applied *in silico* by a virtual screen of over 11,000 fragments on 152 protein-binding sites. This work demonstrated that a small ligand based virtual screen can be effective at predicting druggability of protein-binding sites [88].

#### Structure-based druggability

Knowing the 3D structure of a target protein greatly assists small molecule drug discovery, enabling analysis of the druggability of each protein pocket, virtual docking with small molecules and comparison of similar proteins [89]. Structure-based druggability calculations starts with a crystallographic or modelled 3D structure. All the ligand-binding sites on the surface of the protein are identified and the probable druggability of each pocket is assessed based on physicochemical parameters such as size, shape and hydrophobicity. Results from these tools correlate well with predictions from NMR screens of fragment libraries [87,90] and drug discovery projects are more likely to fail if they target proteins that have only low scoring pockets [91].

**Figure 2:**
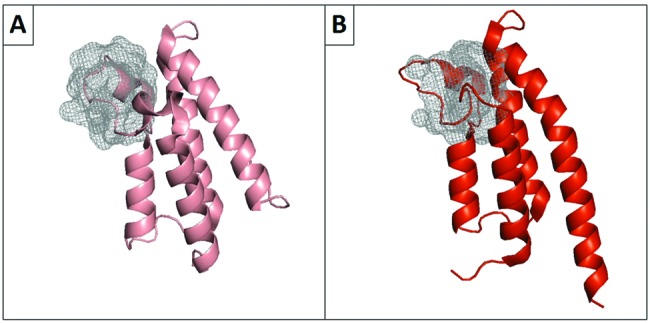
Prediction of druggable pockets in bromodomains The acetyl-lysine (KAc) binding pockets of two human bromodomains were identified by DoGSiteScorer. (**A**) shows the non-druggable KAc binding site of the bromodomain from TRIM24 (PDB: 2YYN_A) (druggability score =0.49). (**B**) shows the druggable KAc binding site of the bromodomain from BRD1 (PDB: 3RCW_A) ( druggability score =0.68). A score greater than 0.50 is indicative of a druggable pocket [102].

Methods for identifying pockets either assume that the 3D protein structure is static, employ energy-based algorithms or use molecular dynamics simulations [92]. These techniques have been reviewed [93–95] and the main algorithms that search for binding pockets have been summarized by Villoutreix and colleagues [95]. The druggability of each pocket is then assessed by calculating properties such as hydrophobicity, volume, amino acid composition and electrostatics, and then using these features to train a machine learning model on validated drug binding/not drug-binding pockets [90,96–99]. Some of these programmes are automated and score binding pockets on the likelihood of their druggability. An overview of these automated druggability assessment methods is summarized in Table 3. Figure 2 demonstrates the potential to drug the bromodomains BRD1 and TRIM24 using DoGSiteScorer. Although the proteins appear indistinguishable to the eye, nevertheless the analysis identifies that BRD1 has a bromodomain-binding pocket (shown as a mesh) more likely to bind a small molecule.

**Table 3:** Programs that can be used to calculate structure-based druggability

Name	References	Pocket search method	Druggability score	
			Function	Descriptors
fPocket	[92]	Geometric criteria based on distance to predetermined points	Partial least square analysis	Hydrophobicity, normalized polarity and local hydrophobicity density
DoGSiteScorer	[100]	Geometric criteria based on 3D image enhancement techniques	Support vector machine	Depth, volume and relative number or apolar amino acids
SiteMap	[101]	Geometric and energetic criteria on 3D grids	Weighted sum of three descriptors	Hydrophilicity, degree of enclosure, number of site points

Understanding the resemblance between binding pockets can aid in the design of target selective compounds, preventing mistakes in assigning druggability. To aid this understanding, a number of tools have been developed that compare protein-binding sites by representing binding sites through specific features [95,103,104]. Computational druggability investigations have also been undertaken to compare and contrast the druggability of binding sites such as bromodomains that have a similar function [105–108].

#### Network-based druggability

**Figure 3:**
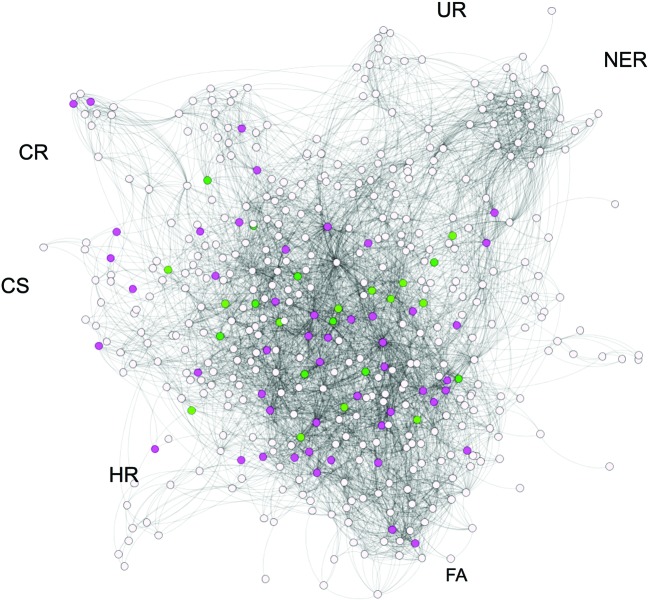
Druggable targets in the DNA damage response This illustrates the protein–protein interaction network of proteins (derived from the STRING database [114]) involved in the DNA damage response as described in [46]. Each protein is shown as a node/circle with the interaction described as a connecting line. The network is labelled by some of the DDR processes: HR; UR, ubiquitin response; FA, Fanconi anaemia; NER, nucleotide excision repair, CS, chromosome segregation; CR, chromatin remodelling. Nodes coloured dark green indicate a protein for which these is a licenced drug, light green nodes indicate that the protein is a target of a drug in clinical trials. Pink nodes indicate that a protein is predicted to be druggable as it has the features of a good drug target. Each of these proteins have been predicted to be druggable by the at least two of the druggability methods (ligand, structure and network) provided by the canSAR database [84]. Adapted from Supplementary Information (figure) S15 [46]. .

A number of different networks have been built to represent molecular interactions including drug–target, drug–drug, drug–disease, protein–protein, transcriptional and signalling networks (for an example, see Figure 3). Features from these networks can then be used to train machine learning models with a large number of aims. These range from characterizing drug targets and identifying potential new uses for existing drugs, to predicting the response of patient subpopulations to drug treatments [109–112]. Similarly, Napolitano et al. [113] used machine learning to predict the therapeutic class of FDA-approved compounds with repositioning in mind.

Computational drug repositioning in this way is only possible because of the breadth of publicly available big data sources that integrate pharmacological, genomic, phenotypic, chemical and clinical information (e.g. Drug Bank [115], ClinicalTrials.gov [116], PharmGKB [117] or PubChem [118]) and contine advances in text mining. Many of these tools and networks rely on the semiautomatic identification of links among genomic data, specific molecular pathways and phenology. The development of gene ontological terms has been of particular importance [119] as have advances in text mining approaches. These have recently been reviewed by Gonzalez et al. [120]. Useful resources for protein–protein and genetic interactions are set out in Table 4.

**Table 4:** Web-based databases documenting protein and genetic interactions

Database	References	Description	URL
BioGRID	Chatr-Aryamontri et al. [121]	Repository of curated genetic and physical interaction data	https://thebiogrid.org
STRING	Szklarczyk et al. [122]	Protein–protein interaction data for a wide range of organisms	https://string-db.org
IntAct	Hermjakob et al. [123]	Molecular interaction database derived from literature curation or direct user submissions	http://www.ebi.ac.uk/intact/
Syn-lethality database	Li et al. [124]	Cross referenced and annotated resource for synthetic lethal related research	http://ntu.edu.sg/home/zhengjie/software/Syn-Lethality
SynLethDB	Guo, Liu and Zheng [125]	Database of genetic interactions focused on selective and sensitive anticancer drug targets	http://histone.sce.ntu.edu.sg/SynLethDB/
Slorth!	Benstead-Hume and Pearl [unpublished]	Genetic interaction data with a focus on orthologues and conserved interactions	http://rails.biochem.susx.ac.uk:4000

## Patient stratification and personalized medicine

Next generation sequencing and other ‘omic’ technologies are enabling better identification of a wide range of diseases, which will eventually lead to targeted therapies and personalized medicines. Personalized medicine can be used not only in cancer and long-term disorders, but also in infectious diseases.

One of the best known examples of patient stratification currently used in the clinic is the analysis of biomarkers for patients with breast cancer. There already exists tests and endocrine therapies for patients testing positive for elevated levels of HER2, oestrogen or progesterone and these complement chemotherapy, radiation therapy and surgery, the current standard-of-care in cancer treatment [126]. This type of characterzation is also being developed for other cancers enabling the identification of patient cohorts with similar therapeutic needs and potential outcomes. This approach can also reduce the use of aggressive therapies where they are not warranted. Hoadley et al. [127] divided the heterogeneous population of tumours into clinically and biologically meaningful subtypes using the similarity of molecular profiles. Rubio-Perez et al. [128] developed a pan-cancer strategy for therapy based on identifying alterations in driver genes. While only 5.9% of the tumours were treatable using approved drugs following the clinical guidelines, up to 40.2% could benefit from repurposing existing drugs [128]. A number of other teams have focused on specific common cancers [129–131]

Patient stratification can also be applied to infectious diseases, where patient response to treatment can have a strong genetic component. The 2009 GWAS of patient response to treatment for the hepatitis C virus (HCV) found that a genetic polymorphism near the *IL28B* gene makes a significant difference to patients’ response to pegylated interferon α plus ribavirin [132]. Genotyping people with HCV is now common when determining treatment options [133].

## Discussion

The large amount of data generated directly by the drug discovery process that have become publicly available (e.g. ChEMBL), combined with the disease-based data provided by large consortia (e.g. GDC) mean that there has been an explosion of computational approaches linking chemical and disease data. Innovative bioinformatics approaches are already having an impact on the discovery, preclinical and clinical phases of the drug discovery process.

However, the challenges faced by the pharmaceutical industry means that it is becoming crucial to further invest in the bioinformatics resources required to support and expedite translational drug discovery. Approaches include: the development of databases and data warehouses that can archive, maintain and integrate large amounts of drug discovery and biomedical data currently being generated; the development of robust algorithms to enable the analysis of large and complex datasets; development of tools to enable experimental drug discovery for scientists to easily access and interpret these data; formal and informal networking tools such as Biostars that enable bioinformaticians to link up and learn from one another [134].

These type of endeavours will enable a better understanding of how we can use genomics and other ‘omic’ approaches to classify disease, improve diagnoses and inform new approaches to drug repositioning. They will allow us to identify disease biomarkers and genetic variants which correlate well with patient outcomes, and use them to improve therapeutic strategies.

## Abbreviations

DDR: DNA damage response
GDC: Genomics Data Commons
GWAS: genome-wide association studies
HCV: hepatitis C virus
HR: homologous recombination
PSA: polar surface area
SSBR: single-strand break repair
SSL: synthetically sensitive or lethal
TCGA: The Cancer Genome Atlas
